# MicroRNA Dysregulation in Cutaneous Squamous Cell Carcinoma

**DOI:** 10.3390/ijms20092181

**Published:** 2019-05-02

**Authors:** Natalia García-Sancha, Roberto Corchado-Cobos, Jesús Pérez-Losada, Javier Cañueto

**Affiliations:** 1 IBMCC-CSIC, Laboratory 7, Campus Miguel de Unamuno s/n, 37007 Salamanca, Spain; nataliagarciasancha@usal.es (N.G.-S.); rober.corchado@usal.es (R.C.-C.); jperezlosada@usal.es (J.P.-L.); 2 Instituto de Investigación Biomédica de Salamanca (IBSAL), Hospital Universitario de Salamanca, Paseo de San Vicente 58-182, 37007 Salamanca, Spain; 3 Departamento de Dermatología, Hospital Universitario de Salamanca, Paseo de San Vicente 58-182, 37007 Salamanca, Spain

**Keywords:** miRNAs, cutaneous squamous cell carcinoma, skin cancer

## Abstract

Cutaneous squamous cell carcinoma (CSCC) is the second most frequent cancer in humans and it can be locally invasive and metastatic to distant sites. MicroRNAs (miRNAs or miRs) are endogenous, small, non-coding RNAs of 19–25 nucleotides in length, that are involved in regulating gene expression at a post-transcriptional level. MicroRNAs have been implicated in diverse biological functions and diseases. In cancer, miRNAs can proceed either as oncogenic miRNAs (onco-miRs) or as tumor suppressor miRNAs (oncosuppressor-miRs), depending on the pathway in which they are involved. Dysregulation of miRNA expression has been shown in most of the tumors evaluated. MiRNA dysregulation is known to be involved in the development of cutaneous squamous cell carcinoma (CSCC). In this review, we focus on the recent evidence about the role of miRNAs in the development of CSCC and in the prognosis of this form of skin cancer.

## 1. Introduction

Cutaneous squamous cell carcinoma (CSCC) is the second most frequent cancer in humans, and its incidence is increasing and underestimated [1,2]. While it usually displays a benign clinical behavior, CSCC may be locally invasive and metastatic. It has demonstrated an epidemiological rise over the past three decades [3], and its incidence is set to double by 2030 in European countries [4]. Its high frequency means that CSCC is responsible for many deaths—a similar number in some areas of the US to those resulting from melanoma and oropharyngeal carcinoma [5]. CSCC is an epidermal keratinocyte-derived, non-melanoma skin cancer that may be influenced by several factors, of which chronic sun exposure is the most important and well known, while older age, fair skin, immunosuppression and previous actinic keratosis are also significant [1].

Many pathways are known to be involved in CSCC development. *P53* mutations, induced by ultraviolet radiation [6], are early events in CSCC development and are responsible for severe genomic instability [7]. Indeed, CSCC is the human cancer with the greatest mutational burden [8]. Other suppressor genes, such as *P16* [9], and oncogenes, such as *RAS* [10], are also frequently involved. The accumulation of genetic changes ultimately affects important signaling pathways [11], involving EGFR overexpression, NF-kB activation and NOTCH inactivation [7,12,13,14]. In addition to genetic changes, some critical epigenetic modifications contribute to the process of CSCC carcinogenesis. Although our knowledge of the molecular features of CSCC has grown in recent years, there is still much to be learned.

MicroRNAs (miRNAs or miRs) are endogenous, small, non-coding RNAs of 19–25 nucleotides in length, that are involved in regulating gene expression at a post-transcriptional level [15,16,17]. The long-established central dogma of molecular biology explains the progression from DNA to proteins in cells. Within this conception, miRNAs represented a milestone, since these small molecules of RNA do not translate into proteins and instead exercise their functions in a different manner. MiRNAs regulate the expression of many genes by hybridizing to the 3′-untranslated region (3′-UTR) of target messenger RNAs (mRNAs). Thus, miRNAs block translation or cause mRNA degradation. Target recognition occurs via a 6–8 nt site that matches the miR seed region. Each miR can repress hundreds of genes, each of which can be targeted by multiple miRs, making it a robust system for fine-tuning gene expression [18,19].

**Figure 1:**
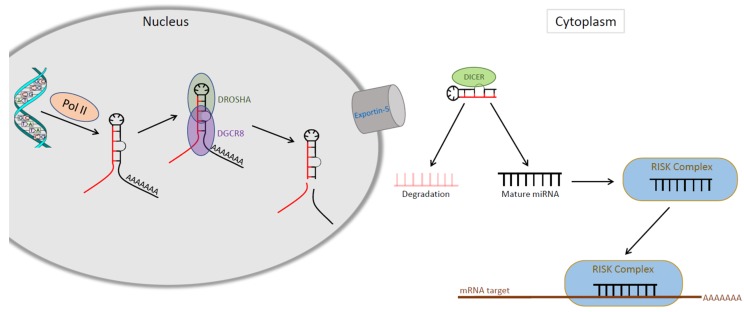
Biogenesis of miRNAs.

Most genes that encode miRNAs localize in intronic, intergenic or antisense regions in the sequences of some genes, and some are located near other miRNAs, supporting the hypothesis that miRNAs can also be transcribed in clusters [19,20]. The biogenesis of miRNAs involves several steps: (i) miRNAs are initially transcribed as long precursor transcripts known as primary miRNAs (pri-miRNAs) by RNA polymerase II (pol II); (ii) pri-miRNAs are processed by the DROSHA/DGCR8 complex to precursor miRNAs (pre-miRNAs); (iii) pre-miRNAs are exported to the cytoplasm, where the RNAsa-III DICER enzyme processes the pre-miRNA to generate a double-stranded RNA molecule; (iv) the duplex unwinds; and (v) the mature miR strand is incorporated into the RNA-induced silencing complex (RISC), which guides target mRNA silencing (Figure 1). The degree of complementarity between the sequences determines whether the target mRNA is degraded or protein translation is repressed [19]. Recent studies have shown that, in contrast to the standard binding of miRNAs to the 3’-UTR region of mRNA, some miRNAs can also bind the 5′-untranslated region (5′-UTR) and open reading frames (ORFs), and determine translational activation and not repression. This occurs under growth-arrest conditions [21]. Mature miRNAs are usually located in the cytoplasm, but are sometimes situated in the nucleus, in the mitochondria, or in small vesicles, performing the important non-canonical roles of miRNAs [21]. In addition, miRNAs are regulated at various levels, including miRNA transcription, RISC blinding, methylation and miRNA decay [22].

**Figure 2:**
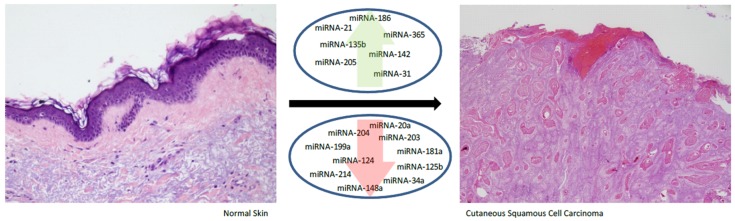
MiRNA dysregulation in cutaneous squamous cell carcinoma compared to normal skin.

MicroRNAs have been implicated in a wide variety of biological processes, including differentiation, proliferation, survival, and apoptosis, as well as immune modulation, inflammation, metabolic control and development [23]. In cancer, miRNAs can proceed as oncogenic miRNAs, promoting carcinogenesis, (oncomiRs) [24] or tumor suppressor miRNAs, preventing cancer development, (oncosuppressor-miRs) depending on the pathway in which they are involved [24,25]. Dysregulation of miRNA expression has been noted in most of the tumors evaluated, such as those of breast, colorectal, ovarian and lung cancers, melanoma, head and neck squamous cell carcinoma and many types of leukemia [26,27,28]. MiRNA dysregulation is known to be involved in the development of cutaneous squamous cell carcinoma (CSCC). This review focuses on the recent findings about the role of miRNAs in the development of CSCC and in the prognosis of this form of skin cancer. First, we consider the miRNAs that exert oncogenic functions in CSCC and later we consider those that act as suppressors. We discuss the prognosis of CSCC depending on miRNAs, and finally, draw attention to possible new research options in the field. Table 1 and Figure 2 show a summary of the miRNAs that are dysregulated in CSCC.

## 2. Onco-miRNAs Involved in Cutaneous Squamous Cell Carcinoma

### 2.1. MicroRNA-21

MiRNA-21 is a well-established oncogenic miRNA that is overexpressed in several human cancers [28,52,53,54,55]. It is the most commonly upregulated miRNA in solid and hematological malignancies [56]. Functional studies in cancer cell lines indicate that miR-21 plays an essential role in oncogenesis, as indicated by its association with high proliferation, low apoptosis, high invasion, and metastatic potential [56,57,58,59,60,61,62]. Essentially, miR-21 functions as an anti-apoptotic and pro-survival factor [63]. The induction of miR-21 is also associated with cellular de-differentiation.

MiR-21 operates by targeting several genes [64], such as *TPM1*, *TIMP3*, *SPRY1*/2, *BCL2, BTG2, CDC25A*, *PTEN*, *PDCD4* [60,65,66,67,68,69,70,71], *FBXO11* [72], and *IGFBP3* [63].

MiR-21 downregulates the expression of two tumor suppressors, *PDCD4* and *PTEN,* in the CSCC cell line, A-431. The inhibition of miR-21 suppresses tumor growth and invasion, and such inhibition exerts proapoptotic functions in CSCC cells [29]. In another study, mice that lacked the *Grhl3* gene in keratinocytes, a potent suppressor of CSCC, displayed susceptibility to chemically induced and spontaneously developed CSCC [30]. *GRHL3* is known to activate *PTEN* transcription by binding to a conserved site in the *PTEN* promoter. PTEN functions by negatively regulating signaling in the phosphatidylinositol-3-kinase pathway, specifically by dephosphorylating PIP3 to prevent activation of AKT and mammalian target of rapamycin (mTOR), thus inhibiting cell survival and proliferation [73]. MiR-21 induces the loss of *PTEN* and *GHRL3*, resulting in the activation of the PI3K/AKT/mTOR and repression of the RAS/MAPK/ERK signaling pathways, and finally, the induction of aggressive CSCC [30]. The inhibition of miR-21 by an antagomir could provide a novel treatment option for CSCC, and levels of miR-21 may have diagnostic and prognostic value [74].

### 2.2. MicroRNA-205

MiRNA-205 has a dual function in cancer, depending on the cell type and tissue context [75], whereby it may function as an oncogene or a suppressor gene. It has been implicated in several forms of cancer, operating through different mechanisms. On the one hand, it helps in the inhibition of the epithelial-to-mesenchymal transition, acting as a suppressor in this context [31,76], while it may also promote invasion, acting as an oncogene [77,78,79].

MiR-205 targets several genes, including *PTEN* [75] and *SHIP2* [80], as tumor suppressors; *HER3* [81], *E2F1*, *E2F5* and *PKCε* [82] as oncogenes; *ZEB1* and *ZEB2* [31] as pro-metastatic genes; and *VEGF-A* [83] as an angiogenic factor.

MiRNA-205 is upregulated in CSCC relative to normal skin [32], in which its expression is restricted to the basal cell layer of progenitor cells, and absent from suprabasal layers. MiR-205 maintains epithelial proliferation during skin development, helping to maintain skin stemness, and the lack of expression of this miRNA inhibits the proliferation of cells in the basal layer [84]. While miR-205 represses the epithelial-to-mesenchymal transition through the inhibition of ZEB factors [31], it is prominent along the front of invasion [85]. MiR-205 promotes keratinocyte migration by targeting *SHIP2.* SHIP2 is a ubiquitous lipid phosphatase that dephosphorylates PIP3 to modulate AKT signaling in keratinocytes. MiR-205 enhances AKT-signaling pathways via SHIP2 suppression, leading to improved cell survival [33]. MiR-205 is significantly upregulated in invasive cells, compared with in situ CSCCs [86], and is more frequently expressed in CSCCs with high-risk histopathological features [85], all of which suggest an oncogenic role for miR-205 in CSCCs. Its expression enables prognostic CSCC subgroups to be identified [85].

### 2.3. MicroRNA-365

MiR-365 plays crucial roles in tumor progression in several types of human cancer [87,88,89], but acts as a tumor suppressor in other cancers [90,91,92,93]. Its effect depends on the cellular microenvironment and the specific tumor type; it alters proliferation, apoptosis, migration, and invasion in vitro and in vivo [94,95,96].

This miRNA is known to affect various targeted genes, such as *NF-I/B* [97], *BAX* [98], *Cyclin D1* and *BCL-2* [90], *FOS*, *EZH2* and *MCL-1* [93], and *PIK3R3* [99,100].

In a study of the miRNA expression profile in the NIH 3T3 cell line, after irradiation with UVB, miR-365 was found to be an miRNA with extremely high sensitivity to ultraviolet irradiation, which is the most important cause of skin cancer [101]. The overexpression of pre-miR-365-2 in the normal skin cell line HaCaT results in increased proliferation, migration, and invasion in vitro, and cancer cell formation and induction of subcutaneous tumors in BALB/c-nude mice. Moreover, after transfection of anti-miR-365 oligonucleotides in A-431 cells, there is a G1 phase arrest and an increase in apoptosis [34]. MiR-365 downregulates *HOXA9*, which plays an anti-carcinogenic role, inhibiting cell proliferation and promoting cell apoptosis in CSCC. Loss of HOXA9 upregulates HIF-1α, which helps regulate hypoxia response, glucose metabolism and tumor progression [35].

### 2.4. MicroRNA-31

MiR-31 plays a critical regulatory role in embryonic implantation, vascular development, bone and muscle homeostasis, and autoimmunity [102]. MiR-31 expression is reduced in some cancers, such as triple-negative breast, gastric, prostate and bladder cancers [103,104,105,106], but increased in many others, such as colorectal, lung, basal-like breast cancers and head and neck squamous cell carcinoma [107,108,109,110]. The functional role of this miRNA is exceptionally complex because it can act as an oncogenic or a tumor suppressor miR.

Functional studies have demonstrated that miR-31 has multiple target genes that are involved in the cell cycle, DNA repair, metabolism, apoptosis, the chemokine signaling pathway and chemical resistance [111,112].

Recent studies using miRNA arrays have shown that microRNA-31 is overexpressed in CSCC. MiR-31 is overexpressed later, during tumorigenesis, when the lesions are invasive. Experiments with the CSCC cell line UT-SCC-7 showed that inhibition of endogenous miR-31 suppresses cell motility and colony-forming ability [36]. Lin et al. identified *RhoBTB1*, a member of the Rho family of small GTPases, which acts as a tumor suppressor, and whose transcripts are a direct target of miR-31 in CSCC. Experiments involving silencing by siRNA or knockdown in the A-431 cell line indicated that the suppression of *RhoBTB1* by miR-31 induces cell proliferation and invasion [37].

### 2.5. MicroRNA-186

Recent studies suggest that miR-186 is associated with various diseases, such as solid tumors, hematopoietic malignancies, bone disorders and vascular disease [113,114,115,116,117,118,119]. In lung adenocarcinoma, miR-186 downregulation is linked to poor survival [113], and its overexpression suppresses cell proliferation and metastasis [120]. In cancers of the bladder [115] and prostate [121], miR-186 also acts as a tumor suppressor. However, miR-186 promotes pancreatic [122] and endometrial [123] carcinogenesis, by playing an oncogenic role. It has also been implicated in drug sensitivity [124,125].

Downstream target genes of miR-186 such as *HIF1α* [114], *MAP3K2* [120], *Cyclin D1*, *CDK2* and *CDK6* [113], *Twist1* [125], *FOXO1* [123], and *ROCK1* [126], have been tentatively proposed as being involved in a range of cell processes such as cell cycle, EMT and migration.

Apoptosis protease activating factor-1 (*APAF1*) acts as a target gene of miR-186 in A-431 CSCC cells [38]. APAF1 is a key molecule in the intrinsic pathway of apoptosis, which oligomerizes in response to cytochrome *c* release and forms the apoptosome [127]. Its downregulation as a consequence of miR-186 upregulation promotes cell proliferation, invasion and migration, and inhibits cell apoptosis [38].

### 2.6. MicroRNA-142

The biological role of miR-142 is poorly understood, but this miRNA is known to be preferentially expressed in cells of hematopoietic origin, and miR-142 null mice present abnormal lymphopoiesis and immunodeficiency [128,129]. In cancer, miR-142 can act as a suppressor, inhibiting cell proliferation [130,131,132,133], or as an oncogenic miRNA, promoting cellular proliferation and migration [134,135], depending on the type of tumor and the mature sequence generated from the 5′ or 3′ arm of miR-142.

Numerous target genes of microRNA-142 have been described, such as *PD-L1* [133], *BTG3* [136], *CDK4* [137], *RAC1* [138], *MLL1* [132], and *PTEN* [39].

In CSCC, microRNA-142-5p acts as an oncogene. It is more strongly expressed in CSCC cell lines such as A-431, SCC13, HSC-5, and HS-1 than in the normal skin cell line, HaCaT. Its downregulation inhibits CSCC progression, and its upregulation promotes the opposite effect, inducing CSC-like phenotypes through the WNT signaling pathway [39]. WNT/β-catenin-dependent and -independent pathways are frequently hyperactivated in CSCC and promote cell proliferation and invasion [139].

### 2.7. MicroRNA-135b

The miRNA-135b function has been described as being oncogenic [140,141,142] or tumor suppressive [143] in malignant tumors originating from different tissues. It has been implicated in survival, motility, invasiveness, apoptosis, and sensitivity to chemotherapy [144,145].

*APC* [142], *HIF1α* [146], *LZTS1* [141], *LATS2* [140], *FOXO1* [147], *ERα*, *AR* and *HIF1AN* [148] have been reported to be direct targets of miR-135b.

In head and neck SCC and CSCC, miR-135b is a tumor promoter that stimulates cancer cell proliferation, colony formation, angiogenesis [146], migration and invasion [40]. MiR-135b is upregulated in CSCC in immunocompromised patients and organ transplant recipients by modulating *LZTS1*, a tumor-suppressor gene [40]. LZTS1 protein expression is critical for normal mitosis progression, guaranteeing an adequate Cdk1 activity during M phase. Its downregulation shortens the mitotic division time causing improper chromosome segregation [149].

Other miRNAs that are overexpressed explicitly in CSCC include miR-346, miR-17-92, and miR-497. These are involved in angiogenesis, colony formation, migration, invasion, and indicate malignant progression [150,151,152].

## 3. Tumor Suppressor MiRNAs Involved in CSCC

### 3.1. MicroRNA-34a

The miR-34 family consists of three members, miR-34a, miR-34b, and miR-34c, which have suppressive roles in various types of human cancer, including melanoma, prostate, pancreatic, colorectal, ovarian and neuroblastoma [153]. They suppress tumor growth and metastasis by inhibiting the processes that promote cancer development, including cell cycle, EMT and stemness, and by promoting processes that inhibit carcinogenesis, such as apoptosis and senescence [154].

By 2014, 77 miR-34 targets had been validated [154]. MiR-34a regulates multiple targets involved in tumorigenesis and cancer progression, such as *MYC*, *MET*, *CDK4/6*, *NOTCH1*, and *BCL2*, among others; and its expression is controlled by p53 [155].

In the case of CSCC, miR-34a was significantly downregulated in CSCC tissues relative to the adjacent non-tumor tissue. The same pattern of expression was observed in CSCC lines such as A431 and SCL-1 compared with that in HaCaT cells of normal skin. Furthermore, a low level of expression of miR-34a was associated with the aggressive progression of CSCC, whereas the restoration of miR-34a levels in the SCL-1 cell line suppresses proliferation, migration, and invasion [41]. Lefort et al. also described a significant downmodulation of miR-34a in the keratinocyte-derived SCC cell line and tumors [42]. It predicts that a High-mobility group box 1 *(HMGB1)* [41] and Sirtuin 6 (*SIRT6*) [42] are target genes of miR-34a in CSCC. HMGB1 is a non-histone, nuclear-binding protein that participates in the regulation of DNA organization and gene transcription [41]; and SIRT6 is a highly specific NAD+-dependent histone deacetylase and ADP ribosyl transferase that has been implicated in DNA repair, genomic stability and telomere structure [156]. Moreover, miR-34a is induced with differentiation and keratinocyte differentiation, putting the brakes on tumor development [42]. These studies suggest that miR-34a is a tumor suppressor in CSCC, and techniques to overexpress or replace it could provide a basis for valuable therapeutic tools. Indeed, MRX34, which restores the suppressor function of endogenous miR-34, was the first microRNA mimic to be used in a clinical setting [157,158].

### 3.2. MicroRNA-125b

The miR-125 family is involved in a variety of solid carcinomas, hematological malignancies and other diseases, as a repressor or a promoter [159]. For example, microRNA-125a inhibits proliferation and invasion, and facilitates lung cancer cell apoptosis [160]. MicroRNA-125b is overexpressed in colorectal tumors [161], but in contrast, is downregulated in ovarian cancer and head and neck squamous cell carcinoma [28,162].

MicroRNA-125b is one of the most misregulated microRNAs in cancer. Many of its targets have been implicated in a range cellular processes, the most important being *BCL2*, *MMP13*, *IGFR1*, *STAT3*, *CDK6*, *ERBB2/3*, and *c-JUN* [163].

In CSCC, microRNA-125b is downregulated relative to healthy skin. The overexpression of miR-125b in two human CSCC lines (UT-SCC-7 and A-431) suppresses cell proliferation, migration, and invasion and increases the percentage of cells arrested in G1 phase [43]. Matrix metallopeptidases *MMP13* and *MMP7* and mitogen-activated protein kinase 7 (*MAP2K7*) have been identified as targets of miR-125b using bioinformatic analyses. These genes play essential roles in EMT, cancer cell migration, cell growth, inflammation and angiogenesis [43]. These findings support the tumor-suppressive role of miR-125b in CSCC.

### 3.3. MicroRNA-181a

The MiR-181 family is highly conserved, and its members are involved in the development, function, and pathogenesis of immune cells [164]. MiR-181a is involved in diverse cellular functions such as growth, proliferation, death, tumor suppression, carcinogenesis, and drug resistance [165,166,167].

Many target genes of microRNA-181a have been identified, for example, *EGR1* [168], *SMAD7*, *BMPR2*, and *TGFBR1* [169,170], which are involved in the TGF-β pathway.

MiR-181a is downregulated in CSCC compared with in healthy skin [44]. Moreover, HaCaT miR-181a knockdown cells exhibit an increase in viability and form cysts when they are injected subcutaneously into nude mice. Conversely, SCC13 cells with high levels of tetracycline-induced miR-181 attenuate cancer development in vivo and in vitro. They also reveal that miR-181a mediates its tumor-suppressive role through KRAS signaling via the MAPK pathway. Finally, they noted an increase in miR-181a levels during keratinocyte differentiation, suggesting that miR-181 is essential for the transition of keratinocytes into CSCC [44].

### 3.4. MicroRNA-148a

Aberrant expression of the miR148 family has been observed in tumor and non-tumor diseases [171,172]. MiR-148a is underexpressed in many types of cancer [173,174,175], including CSCC [45]. The miR-148a gene activates DNA hypermethylation mechanisms that silence a large number of CpG islands in the promoter and many tumor mechanisms [176,177,178].

The target genes of miR-184a identified in cancer are *USP4* [179], *ROCK1* [180], *ERBB3* [181], *BCL-2* [182], *WNT10B* [183], and *CDC25B* [184].

MAP3K4 and MAP3K9, upstream activators of the JNK and ERK pathways, are target genes of miR-148a in CSCC [45]. Furthermore, the overexpression of miR-148a significantly inhibits metastasis and the inhibition of MAP3K4 or MAP3K significantly decreases cell proliferation [45]. Together, these findings suggest that low levels of miR-148a or high levels of its target genes may be potential predictors of CSCC, making this a putative target for the treatment.

### 3.5. MicroRNA-20a

MicroRNA-20a belongs to the miR-17-92 cluster, which includes six microRNAs: miR-17, miR-18a, miR-19a, miR-19b-1, miR-20a, and miR-92a-1 [185]. Like other miRNAs, miR-20a can function as a tumor suppressor [186,187] or as an oncogene [188,189,190], depending on the type of tumor and context in question.

Some of the target genes of microRNA-20a described are *THBS2* [190], *RUNX3* [191], *CELF2* [189], *PDCD6* [192], *ATG7* [193], *TIMP2* [194], and *LIMK1* [46].

MiR-20a plays a role as a tumor suppressor in CSCC. The level of MiR-20a is significantly lower in CSCC, and the expression of *LIMK1*, a target gene of miR-20a and a known tumor metastasis promoter, is higher in CSCC than in normal skin. The overexpression of miR-20a through plasmids in A-431 and SCL-1 lines inhibits cell proliferation, colony formation, cell migration, and invasion, and raises levels of LIMK1 [46]. LIMK1 is activated via phosphorylation downstream of Rho/Rac/Cdc42 signaling. The substrates of LIMK1 are members of the acting depolymerizing factor (ADF) and cofilin family. The phosphorylation of LIMK1 results in the inactivation of cofilin and the subsequent stabilization of actin filaments, formation of stress fibers and cell invasion [195]. Thus, lower levels of expression of miR-20a could predict poor prognosis of cutaneous squamous cell carcinoma [196], and the upregulated expression of miR-20a could be of therapeutic value.

### 3.6. MicroRNA-203

MiRNA-203 usually acts as a tumor suppressor [197,198,199,200,201,202,203,204,205,206,207], although it has been suggested that it may exhibit oncogenic behavior in some tumors [208,209]. MiRNA-203 is restricted to epithelial tissues and expressed at a higher level in skin than in any other organ [210]. Several independent studies have shown that miR-203 is involved in skin morphogenesis and promotes epidermal differentiation by repressing stemness of keratinocytes [206,211].

MiRNA-203 acts through interaction with several genes, of which the most relevant is *c-MYC* [47]. It also targets *P63*, *LASP1*, *RAN* and *RAPH1* [210], *BMI1* [212], and versican [213].

MiR-203 is downregulated in poorly differentiated CSCCs, and its overexpression suppresses migration, angiogenesis, and invasion in UT-SCC-7 and A-431 cell lines and immune-deficient NOD/SCID gamma mice [47]. MiRNA-203 was shown to exert this function by targeting the proto-oncogene c-MYC and thus, the authors proposed that the miR-203/c-MYC axis presents a potential candidate target for CSCC treatment [47]. MiR-203 downregulates *p63* and thus controls the p63-dependent proliferative potential of epithelial precursor cells during keratinocyte differentiation and epithelial development. MiR-203 may repress suprabasal p63 and restrict cell proliferation in differentiating keratinocytes, acting as a tumor suppressor [214]. Our group described that miR-203 was more frequently expressed in squamous non-malignant cell lines than in malignant groups. Moreover, they showed that miR-203 was more frequently expressed in differentiated rather than in undifferentiated areas in CSCC. They identified miR-203 expression as a feature of CSCC with a better prognosis [85], and it exhibits an opposite pattern of expression in CSCC with miRNA-205 [85].

### 3.7. MicroRNA-204

MicroRNA-204 is an intronic miRNA located at the *TRPM3* gene. Therefore, its expression is under the control of the *TRPM3* promoter, which is frequently methylated in cancer [215]. This location is a cancer-associated genomic region where loss of heterozygosity is very frequent [48]. Studies have demonstrated that miR-204 functions principally as a tumor suppressor gene [215,216,217,218], but with dual activity: in prostate cancer as a tumor suppressor in adenocarcinoma cells and as an oncomiR in neuroendocrine-like cancer [219]

Some target genes of miR-204 have been validated in cancer cells, such as *BCL2* in cholangiocarcinoma, colon cancer and neuroblastoma, *BNDF* and *JAK 2* in breast cancer, *Cyclin D2* in retinoblastoma, *EPHB2* in glioma, *IGFPB5* in papillary thyroid carcinoma [220], and *SHP2* in cutaneous squamous cell carcinoma [48].

MicroRNA-204 is downregulated in CSCC relative to actinic keratosis. DNA methylation of the *TRPM3* promoter region upstream of miR-204 is identified as one of the repressive mechanisms of miR-204 silencing in CSCC. Downregulation of miR-204 results in STAT3 activation and negative MAPK pathway regulation. Nuclear STAT3 signal is observed in CSCC and adjacent actinic keratosis, although in non-peritumoral actinic keratosis, STAT3 activation occurs in the plasma membrane and cytoplasm [48]. STAT3 acts as a transcription factor in nuclei and promotes malignant progression [221]. MiR-204 overexpression could inhibit STAT3 activation and its translocation to the nuclei, with consequent inhibition of carcinoma progression [48].

### 3.8. MicroRNA-199a

MicroRNA-199a is expressed in a broad array of tissues, including the brain, liver, vascular and visceral smooth muscle, ovarian and testicular tissue, cardiomyocytes and endothelial cells [222]. In cancer, it can inhibit tumorigenesis [223,224] or promote tumor growth [225]. Additionally, it has been implicated in drug sensitivity [226,227].

Numerous target genes of microRNA-199a have been described, including *CD44* [49,228], *ROCK1* [229], *HIF1α* [224], and *BCAM*, *FZD6* and *DDR1* in CSCC [50].

Recent data have revealed that miR-199a is downregulated in human CSCC cancer tissue relative to normal tissues. MiR-199a targeted *CD44* to repress the proliferation, migration, and invasion of CSCC cell lines [49]. Moreover, miR-199a regulates the interaction between CD44 and Ezrin, a complex implicated in metastasis [230]. CD44 is a non-kinase transmembrane proteoglycan that mediates its effects on cancer cells by activating signaling pathways including protein kinases, by activating transcription factors and by modulating the cytoskeletal architecture [231]. The downregulation of miR-199a is also associated with the increased activity of MMP2 and MMP9, which are important in EMT [49]. Therefore, miR-199a and its targets might provide the basis for a therapeutic strategy in CSCC.

### 3.9. MicroRNA-124

MicroRNA-124 is the most abundant miRNA in the brain, and it has a central role in nervous system disorders [232]. However, it is also involved in cancer, in which it plays a putative role as a tumor suppressor [233,234,235,236,237].

Some target genes of miR-124 have been described, such as *ROCK1* [238], *SNAIL2* [233], *CDK4* [239], *CAV1* [240], and *ERK2* [51], whose action can suppress cancer growth and metastasis.

In CSCC, miR-124 is downregulated in CSCC compared with seborrheic keratosis tissue and the human CSCC cell line. The downregulation of microRNA-124 mediates abnormal cell proliferation via the induction of ERK2 [51]. ERK2, with ERK1, are key protein kinases that contribute to the Ras-Raf-MEK-ERK MAP kinase-signaling module [241]. Additionally, serum concentration of miR-124 is correlated with miR-124 expression levels in the tumor tissues and inversely correlated with tumor progression [51], suggesting that microRNA-124 could be a biomarker for early detection of cutaneous squamous cell carcinoma.

### 3.10. MicroRNA-214

MicroRNA-214 is an important microRNA in neurogenesis [242], but also acts in tumors as a tumor suppressor [243,244,245] or oncogene [246,247]. The functions of miR-214 depend on the targets and signaling.

MiR-214 targets validated in cancer include *TFAP2* and *ITGA3* in melanoma, *ING4* in pancreatic cancer, *PTEN* in stomach cancer, *LZTS1* in osteosarcoma, *PTEN* and *P53* in ovarian cancer, and *ERK1/2* in cutaneous squamous cell carcinoma [51,248].

In CSCC, microRNA-214 acts as a tumor suppressor miRNA, and it is downregulated in vivo and in vitro. The downregulation of microRNA-214 induces ERK1 and ERK2 [241], which are essential for cellular proliferation, differentiation, and survival; and transfection of the miR-214 mimic reduced the expression of both [51]. Taken together, levels of miR-124/214 and its targets ERK1 and ERK2 could be a tool for early diagnosis and treatment.

## 4. MicroRNAs and Cancer Therapy

The functionality of miRNAs in controlling gene expression in cancer makes miRNAs ideal candidates for targeted therapies. Sandwich RNAi inhibition strategy, which consists of a combination of miRNA guided to an oncogene and siRNA guided to a microRNA, and multiplex RNAi inhibition strategy, in which multiple molecular defects accumulated in a multistep pathway of a specific cancer can be targeted with siRNA, are two approaches described for the treatment of cancer using RNAi. Current strategies for miRNA-inhibition are based on antisense oligonucleotides targeting miRNAs (AMOs), on locked nucleic acid (LNA) antimiRs, on antagomirs, on miRNAs sponges that contain multiple tandem binding sites to a miRNA and on small molecule compounds (SMIRs). To restore miRNAs levels, small molecules have been developed that induce miRNA expression, both miRNA mimics and miRNA expression vectors [249]. To date, most of these therapies have been validated in vitro but more studies are necessary to be able to use them on human patients.

## 5. Conclusions and Future Perspectives

Cutaneous squamous cell carcinoma is the second most frequent cancer in humans. While it usually exhibits a benign clinical behavior, it can be both locally invasive and metastatic to distant sites. In recent years, research efforts have been directed towards deciphering the pathogenic basis of this tumor, but there is much still to be discovered. MicroRNAs are small molecules of non-coding RNA associated with the development of cancers, including CSCC. Several miRNAs are dysregulated in CSCC, exhibiting oncogenic functions (such as mir-21, mir-205, mir-365, mir-31, mir-135b, mir-142, and mir-186) or suppressor functions (such as mir-20a, mir-203, mir-181a, mir-125b, mir-34a, mir-148a, mir-214, mir-124, mir-204, and mir-199a). A better knowledge of miRNAs might shed light on the biology of CSCC and suggest novel molecular targets for the treatment of this disease.

MicroRNA profiling has been useful in identifying predictive miRNA signatures associated with tumor growth, progression and prognosis. Changes of specific miRNAs can be detected using different traditional techniques, such as Northern Blot, reverse transcription qPCR, microarray approaches, next-generation sequencing and in situ hybridization [250]. In recent years, newly incorporated methods, based on nanotechnology and enzymatic amplification, have improved the sensitivity and specificity of miRNA detection [250,251]. In the future, novel technologies will enable us to improve our definition of miRNA biomarkers and to better identify subgroups for prognosis in CSCC.

## Figures and Tables

**Table 1:** MicroRNAs (MiRNAs) implicated in cutaneous squamous cell carcinoma and their functions. Panel A (onco-miRNAs) and Panel B (tumor suppressor miRNAs).

**Onco-miRNAs in CSCC**			
**MiRNA**	**Target Genes**	**Function**	**Ref.**
MicroRNA-21	*PTEN*, *PDCD4*, *GRHL3*	tumor growth, invasion, antiapoptotic	[29,30]
MicroRNA-205	*ZEB*, *SHIP2*	cell proliferation, keratinocyte migration	[31,32,33]
MicroRNA-365	*HOXA9*	cell proliferation, migration, invasion	[34,35]
MicroRNA-31	*RhoBTB1*	migration, invasion	[36,37]
MicroRNA-186	*APAF1*	cell proliferation, migration, invasion, antiapoptotic	[38]
MicroRNA-142	*PTEN*	CSCC progression, maintenance stem cell properties	[39]
MicroRNA-135b	*LZTS1*	cell proliferation, migration, invasion	[40]
**Tumor suppressor miRNAs in CSCC**			
**MiRNA**	**Target genes**	**Function**	**Ref**
MicroRNA-34a	*HMGB1*, *SIRT6*	cell proliferation, migration, invasion	[41,42]
MicroRNA-125b	*MMP13*, *MMP7*, *MAP2K7*	cell proliferation, migration, invasion, inflammation, angiogenesis	[43]
MicroRNA-181a	*KRAS*	survival	[44]
MicroRNA-148a	*MAP3K4*, *MAP3K9*	metastasis	[45]
MicroRNA-20a	*LIMK1*	cell proliferation, colony formation, migration, invasion, metastasis	[46]
MicroRNA-203	*c-MYC*	migration, angiogenesis, invasion	[47]
MicroRNA-204	*SHP2*	CSCC progression	[48]
MicroRNA-199a	*CD44*, *BCAM*, *FZD6*, *DDR1*	cell proliferation, migration, invasion, metastasis	[49,50]
MicroRNA-124	*ERK2*	tumor progression	[51]
MicroRNA-214	*ERK1*, *ERK2*	cell proliferation, differentiation, survival	[51]
